# CYP2C19 expression modulates affective functioning and brain anatomy – a large single-center community-dwelling cohort study

**DOI:** 10.1192/j.eurpsy.2022.333

**Published:** 2022-09-01

**Authors:** C. Grosu, O. Trofimova, M. Gholam, M.-P. Strippoli, F. Kherif, A. Lutti, M. Preisig, B. Draganski, C. Eap

**Affiliations:** 1 Lausanne University Hospital, Psychiatry, Prilly, Switzerland; 2 Lausanne University Hospital, Psychiatry, Lausanne, Switzerland

**Keywords:** CYP2C19, behavior, Global Assessment of Functioning, Hippocampus

## Abstract

**Introduction:**

The association between CYP2C19 poor metabolizer status, depressive symptom severity and hippocampal volume in humans is controversial. Progress in understanding not only the pathophysiology of depression but also potential protective mechanisms is important both for daily clinical practice and for the development of new antidepressant therapies.

**Objectives:**

To test and validate previous findings regarding the impact of CYP2C19 status on depressive symptoms and to examine whether it could influence hippocampus subregions and brain tissue microstructure.

**Methods:**

A total of 4152 individuals from the Longitudinal cohort in the community-dwelling adult population - Colaus|PsyCoLaus in Lausanne, Switzerland were included. They have participated in at least one psychiatric evaluation. Brain anatomy patterns using a comprehensive set of psychometry, water diffusion- and relaxometry-based magnetic resonance imaging data were analysed in a subset of the cohort (BrainLaus, n=1187).

**Results:**

In this population-based cohort study, better lifetime global assessment of functioning scores were observed in poor metabolizers when compared to other metabolizers, this result was mainly driven by female participants (ß=3.9, P=0.01). Examination of brain imaging data revealed that higher right hippocampal subiculum volume was related to poor metabolizer status (ß=0.03, P=0.006). In addition, associations were observed between metabolizer status and white matter microstructure in the left uncinate fasciculus (ß=-0.01, P=0.01) and the left cingulum bundle (ß=-0.01, P=0.01).

**Conclusions:**

CYP2C19 status is associated with modifications in lifetime global functioning, and brain anatomy. Such differences in brain structures can contribute to explain the protective effect of CYP2C19 poor metabolizer status.

**Disclosure:**

No significant relationships.

